# Genetic analysis implicates *ERAP1* and HLA as risk factors for severe Puumala virus infection

**DOI:** 10.1093/hmg/ddae158

**Published:** 2024-11-13

**Authors:** Hele Haapaniemi, Satu Strausz, Anniina Tervi, Samuel E Jones, Mari Kanerva, FinnGen, Estonian Biobank Research Team, Erik Abner, Anne-Marie Fors Connolly, Hanna M Ollila

**Affiliations:** Institute for Molecular Medicine Finland, FIMM, HiLIFE, University of Helsinki, Tukholmankatu 8, 00290 Helsinki, Finland; Institute for Molecular Medicine Finland, FIMM, HiLIFE, University of Helsinki, Tukholmankatu 8, 00290 Helsinki, Finland; Department of Oral and Maxillofacial Diseases, Helsinki University Hospital and University of Helsinki, Haartmaninkatu 1, 00290 Helsinki, Finland; Department of Plastic Surgery, Cleft Palate and Craniofacial Center, Helsinki University Hospital and University of Helsinki, Stenbäckinkatu 11, 00290 Helsinki, Finland; Broad Institute of MIT and Harvard, 415 Main Street, Cambridge, MA 02142, United States; Institute for Molecular Medicine Finland, FIMM, HiLIFE, University of Helsinki, Tukholmankatu 8, 00290 Helsinki, Finland; Institute for Molecular Medicine Finland, FIMM, HiLIFE, University of Helsinki, Tukholmankatu 8, 00290 Helsinki, Finland; Department of Hospital Hygiene and Infection Control, TYKS Turku University Hospital, Kiinamyllynkatu 4-8, 20521 Turku, Finland; Estonian Genome Centre, Institute of Genomics, University of Tartu, Riia tn 23b, 51010 Tartu, Estonia; Department of Clinical Microbiology, Umeå University, Biologihuset, 90187 Umeå, Sweden; Institute for Molecular Medicine Finland, FIMM, HiLIFE, University of Helsinki, Tukholmankatu 8, 00290 Helsinki, Finland; Broad Institute of MIT and Harvard, 415 Main Street, Cambridge, MA 02142, United States; Department of Anesthesia, Critical Care and Pain Medicine, Massachusetts General Hospital and Harvard Medical School, 55 Fruit Street, GRB 444 Boston, MA, United States; Center for Genomic Medicine, Massachusetts General Hospital and Harvard Medical School, 185 Cambridge Street, Boston, MA 02114, United States

**Keywords:** Puumala virus, GWAS, fine-mapping, HLA

## Abstract

Puumala virus (PUUV) infections can cause severe illnesses such as Hemorrhagic Fever with Renal Syndrome in humans. However, human genetic risk factors contributing to disease severity are still poorly understood. Our goal was to elucidate genetic factors contributing to PUUV infections and understand the biological mechanisms underlying individual vulnerability to PUUV infections. Leveraging data from the FinnGen study, we conducted a genome-wide association study on severe Hemorrhagic Fever with Renal Syndrome caused by PUUV with 2227 cases. We identified associations at the Human Leukocyte Antigen (HLA) locus and *ERAP1* with severe PUUV infection. HLA molecules are canonical mediators for immune recognition and response. *ERAP1* facilitates immune system recognition and activation by cleaving viral proteins into smaller peptides which are presented to the immune system via HLA class I molecules. Notably, we identified that the lead variant (rs26653, OR = 0.84, *P* = 2.9 × 10–8) in the *ERAP1* gene was a missense variant changing amino acid arginine to proline. From the HLA region, we showed independent and significant associations with both HLA class I and II genes. Furthermore, we showed independent associations with four HLA alleles with severe PUUV infection using conditional HLA fine mapping. The strongest association was found with the HLA-C*07:01 allele (OR = 1.54, *P* = 4.0 × 10^−24^) followed by signals at HLA-B*13:02, HLA-DRB1*01:01, and HLA-DRB1*11:01 alleles (*P* < 5 × 10^−8^). Our findings suggest an association of viral peptide processing with *ERAP1* and antigen presentation through HLA alleles that may contribute to the development of severe PUUV disease.

## Introduction

Puumala orthohantaviruses (PUUV) belong to the Hantaviridae family. PUUV primarily circulates among rodent populations establishing persistent infections and transmits to humans through contact with rodent excretions [1]. Of the two hantaviral diseases, Hantavirus Pulmonary Syndrome (HPS) and Hemorrhagic Fever with Renal Syndrome (HFRS), PUUV causes only HFRS which is characterized by symptoms such as fever, kidney dysfunction, and hemorrhagic complications. Head and back pain are common symptoms, and chest X-ray findings and transient ocular myopia may also occur during the acute stage of PUUV [2, 3]. Despite having a low fatality rate, approximately 52% of the symptomatic cases require hospitalization [4] and 5% of the hospitalized patients need dialysis or intensive care treatment for acute kidney injury (AKI) [1]. In addition, PUUV often results in complications and long-term hormonal, renal or cardiovascular consequences [5].

In Finland, PUUV has a significant impact on public health. The reported average incidence rate of diagnosed PUUV infections in Finland is 31–39 cases per 100 000 inhabitants, which is substantially higher than in other European countries [3]. However, the true incidence rate is estimated to be seven to eight times higher based on the seroprevalence rate (12.5% in Finland) [6].

Due to the high incidence of PUUV infections in Finland, much research has been invested in epidemiology, virology, clinical courses, and the ecology of the virus in the carrier bank vole (*Myodes Glareolus*) [1, 3]. The effect of host genetics on PUUV disease susceptibility and severity has primarily been explored through Human Leukocyte Antigen (HLA) haplotype studies. Small case–control studies have identified several HLA alleles and haplotypes associated with severe disease, although the findings vary between studies [7–10].

We utilized data from 520 210 individuals participating in the FinnGen project to explore the biological mechanisms behind PUUV infection. With International Classification of Diseases (ICD) code-based case definition, we identified 3650 cases of PUUV infection diagnosed between 1995 and 2023. Out of these cases, 2227 required hospitalization and were classified as severe. We assessed the host genetic component with Genome-Wide Association study (GWAS) and fine-mapping of the HLA region and explored the comorbidity burden with epidemiological analyses.

## Materials and methods

### Study cohort

***FinnGen*** is a population-based public-private population cohort established in 2017 [11]. The study combines genetic data with electronic health record data, including International Classification of Diseases (ICD) codes spanning an individual’s entire lifespan, derived from primary care registers, hospital inpatient and outpatient visits, drug prescriptions and several other registries. The project aims to improve understanding of the genetic etiology of diseases and disorders potentially leading to drug development. For this study, we leveraged data from FinnGen’s data freeze 12, which includes 520 210 participants.

To identify Puumala virus cases from the cohort ICD10 code A98.5 for Hemorrhagic Fever with Renal Syndrome has been used. Data was derived from hospital and primary care registers. The severe cases were classified as those requiring hospitalization whereas the non-severe cases were not hospitalized.

Total number of all diagnosed PUUV cases in Finland were obtained from the National Registry for Infectious Diseases at the National Institute for Health and Welfare, from publicly available data [12]. All laboratories notify new diagnoses of communicable diseases there.

### FinnGen ethics statement

Patients and control subjects in FinnGen provided informed consent for biobank research, based on the Finnish Biobank Act. Alternatively, separate research cohorts, collected prior the Finnish Biobank Act came into effect (in September 2013) and start of FinnGen (August 2017), were collected based on study-specific consents and later transferred to the Finnish biobanks after approval by Fimea (Finnish Medicines Agency), the National Supervisory Authority for Welfare and Health. Recruitment protocols followed the biobank protocols approved by Fimea. The Coordinating Ethics Committee of the Hospital District of Helsinki and Uusimaa (HUS) statement number for the FinnGen study is Nr HUS/990/2017.

The FinnGen study is approved by Finnish Institute for Health and Welfare (permit numbers: THL/2031/6.02.00/2017, THL/1101/5.05.00/2017, THL/341/6.02.00/2018, THL/2222/6.02.00/2018, THL/283/6.02.00/2019, THL/1721/5.05.00/2019 and THL/1524/5.05.00/2020), Digital and population data service agency (permit numbers: VRK43431/2017-3, VRK/6909/2018-3, VRK/4415/2019-3), the Social Insurance Institution (permit numbers: KELA 58/522/2017, KELA 131/522/2018, KELA 70/522/2019, KELA 98/522/2019, KELA 134/522/2019, KELA 138/522/2019, KELA 2/522/2020, KELA 16/522/2020), Findata permit numbers THL/2364/14.02/2020, THL/4055/14.06.00/2020, THL/3433/14.06.00/2020, THL/4432/14.06/2020, THL/5189/14.06/2020,THL/5894/14.06.00/2020, THL/6619/14.06.00/2020, THL/209/14.06.00/2021, THL/688/14.06.00/2021, THL/1284/14.06.00/2021, THL/1965/14.06.00/2021, THL/5546/14.02.00/2020, THL/2658/14.06.00/2021, THL/4235/14.06.00/2021, Statistics Finland (permit numbers: TK-53-1041-17 and TK/143/07.03.00/2020 (earlier TK-53-90-20) TK/1735/07.03.00/2021, TK/3112/07.03.00/2021) and Finnish Registry for Kidney Diseases permission/extract from the meeting minutes on 4th July 2019.

The Biobank Access Decisions for FinnGen samples and data utilized in FinnGen Data Freeze 10 include: THL Biobank BB2017_55, BB2017_111, BB2018_19, BB_2018_34, BB_2018_67, BB2018_71, BB2019_7, BB2019_8, BB2019_26, BB2020_1, BB2021_65, Finnish Red Cross Blood Service Biobank 7.12.2017, Helsinki Biobank HUS/359/2017, HUS/248/2020, HUS/150/2022 § 12, §13, §14, §15, §16, §17, §18, and §23, Auria Biobank AB17-5154 and amendment #1 (August 17 2020) and amendments BB_2021-0140, BB_2021-0156 (August 26 2021, Feb 2 2022), BB_2021-0169, BB_2021-0179, BB_2021-0161, AB20-5926 and amendment #1 (April 23 2020) and it’s modification (Sep 22 2021), Biobank Borealis of Northern Finland_2017_1013, 2021_5010, 2021_5018, 2021_5015, 2021_5023, 2021_5017, 2022_6001, Biobank of Eastern Finland 1186/2018 and amendment 22 §/2020, 53§/2021, 13§/2022, 14§/2022, 15§/2022, Finnish Clinical Biobank Tampere MH0004 and amendments (21.02.2020 & 06.10.2020), §8/2021, §9/2022, §10/2022, §12/2022, §20/2022, §21/2022, §22/2022, §23/2022, Central Finland Biobank 1-2017, and Terveystalo Biobank STB 2018001 and amendment 25th Aug 2020, Finnish Hematological Registry and Clinical Biobank decision 18th June 2021, Arctic biobank P0844: ARC_2021_1001.

### Genotyping and quality control

The samples of FinnGen were genotyped using Illumina (Illumina) and Affymetrix arrays (Thermo Fisher Scientific). The array consists of 735 145 probes that capture 655 973 variants encompassing core backbone variants essential for imputation, rare coding variants that are enriched in the Finnish population, as well as variants associated with KIR and HLA haplotypes. Additionally, the array includes markers specific to certain diseases and pharmacogenomic markers [11].

To ensure data integrity, genotyping information from prior chip platforms and reference genome builds was lifted over to build 38 (GRCh38/hg38). Rigorous sample-wise quality control measures were implemented, including the exclusion of individuals with discrepancies between genetically inferred sex and reported sex in registries, high genotype missingness (>5%), and excess heterozygosity (±4 standard deviations). Variant-level quality control involved filtering out variants with high missingness (>2%), low Hardy–Weinberg equilibrium (*P* < 1 × 10^−6^), and a minor allele count < 3 [11].

For further refinement, chip-genotyped samples underwent pre-phasing with Eagle 2.3.5, followed by imputation using the Finnish-specific SISu v4 imputation reference panel. Post-imputation quality control criteria included the exclusion of variants with an INFO score < 0.7 [11].

### HLA imputation

HLA imputation in FinnGen was performed for *HLA A, HLA B, HLA C, HLA DRB1, HLA DQA1, HLA DQB1, HLA DPB1, HLA DRB3, HLA DRB4* and *HLA DRB5* using R library HIBAG (HLA genotype imputation with attribute bagging), as described by Ritari et al. [13]. The HLA imputation of FinnGen data is based on a set of SNPs directly genotyped on the FinnGen array. A Finnish reference panel, genotyped at clinical grade accuracy (4-digits, amino acid), was used.

### Genetic analyses

GWAS in FinnGen was conducted using the REGENIE pipeline (https://github.com/FINNGEN/regenie-pipelines) adjusting for age, sex, chip, batch and ten first principal components. The ICD-10 code for hemorrhagic fever with renal syndrome (A98.5), was used to identify individuals with PUUV infection. The cases were further divided to severe and non-severe based on their need for hospitalization. GWAS analyses were performed for severe PUUV infection versus the remainder of the FinnGen participants and for severe PUUV infection versus non-severe PUUV infection. Additional GWAS for sensitivity assessments were performed between PUUV against population control and non-severe PUUV against population control (Table S5). Manhattan plots and Miami plot were created using R version 4.0.1 (packages: qqman [14] and RColorBrewer). Summary statistics for severe PUUV vs population control and Severe PUUV vs mild PUUV are available in GWAS catalog.

Association testing for HLA variants was conducted using multivariate logistic regression to elucidate the relationships between individual HLA alleles and PUUV infection. Multivariate logistic regression analysis was adjusted for age at death or the end of follow-up, sex, and the first 10 genetic principal components accounting for population structure. Multivariate logistic regression was performed in a stepwise manner by sequentially adding the most strongly associated HLA allele as a covariate to the analysis. This iterative process was repeated until no significant (*P* < 0.05) alleles remained. The multivariate logistic regression analyses were conducted using R 4.0.1 and utilized the packages data.table [15], dplyr [16], and tidyverse [17].

### Epidemiological analyses

We conducted logistic regression analysis to explore associations between PUUV and thrombocytopenia (ICD-10: D69.6, D69.59), acute renal failure (ICD-10: N17, N18, N19, N08.1, N16.0, N29.1), thrombosis (ICD-10: I80.1, I80.2, I80.3, I80.8, I80.9, I81.9, I82.2, I82.3, I82.8, I82.9), lymphoid malignancies (ICD-10: C81-C96) and hypopituitarism (ICD-10: E23.0) in FinnGen (Table S1). The model was adjusted for age at death or end of follow-up, sex and the first 10 genetic principal components. Additionally, we constructed a model that was further adjusted for BMI in addition to the covariates mentioned above.

To evaluate the temporal aspect, we employed the Cox proportional hazards model with age as the timescale and sex as a covariate. Prevalent cases, meaning the individuals who had obtained the studied conditions prior to PUUV infection, were excluded from the analysis [18].

### *ERAP1* haplotype analysis

To strengthen our understanding about the role of ERAP1 in PUUV infection we conducted ERAP1 haplotype analysis. ERAP1 haplotype defining variants were identified from paper by Gelfman *et al.* [19]. We extracted the variants using PLINK [20] and constructed Finnish haplotypes from these variants using data from 520 210 Finnish individuals. The haplotypes present in Finland differed significantly to these presented in the paper of Gelfman *et al.*, so instead of using their suggested haplotypes having low prevalences in Finland, we proceeded with ten most common ERAP1 haplotypes present in FinnGen (Table S6).

We studied the association of each haplotype with severe Puumala virus infection using linear regression models and adjusting with age, age^2^, sex, age*sex and ten first principal components. Furthermore, we estimated the correlation between ERAP1 lead variant, rs26653, and ten most prevalent haplotypes using Pearson correlation.

## Results

### Epidemiological analysis shows PUUV infection is associated with thrombocytopenia and acute renal failure

**Figure 1:**
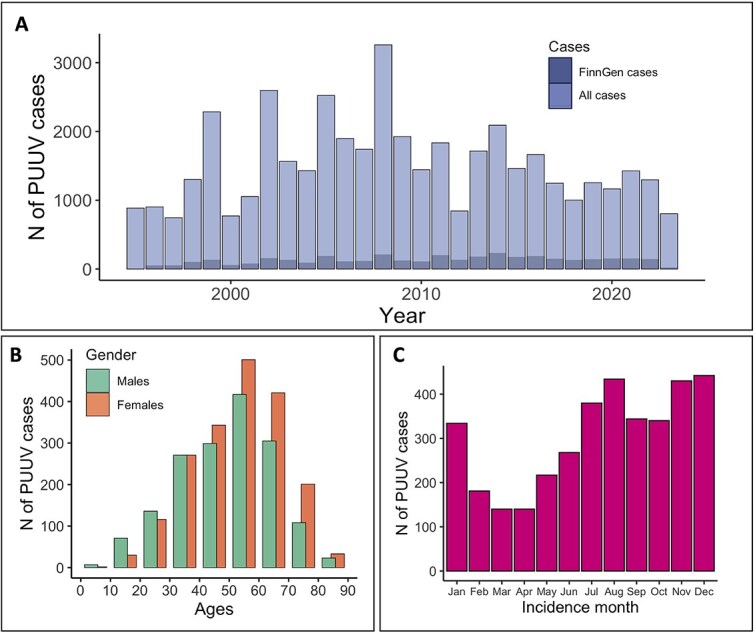
(A) Annual numbers of Puumala virus (PUUV) cases by ICD-10 coding in FinnGen and total numbers of new PUUV diagnoses notified by laboratories in the national infectious diseases registry in Finland. FinnGen has data on about half a million Finns, which corresponds to almost 10% of the total population in Finland. Incidences for all Finns were obtained from the Finnish National Infectious Diseases Register. (B) Age at the first PUUV diagnosis in FinnGen for males and females. (C) Monthly incidence of PUUV in FinnGen.

Using data from FinnGen, we identified 3650 individuals (8.3%) with ICD10 code A98.5-based Hemorrhagic fever with renal syndrome. In Finland, these cases are primarily caused by Puumala hantavirus. We first performed a descriptive analysis of the cohort to understand the associations between PUUV infection and demographic factors. We observed the highest incidence of PUUV infections among people aged 50–60 years (median 52 years) (Fig. 1B). In addition, we saw variation in incidence rates within a year as well as between years. The highest incidence rates were seen in autumn and a peak year occurs every 3–4 years following the changes in vole population as reported previously [21] (Fig. 1A and C). Lastly, we observed an over-representation of males within the cases (54.0%) compared to controls (43.5%) (*P* = 2.4 × 10^−6^) and a slightly higher BMI in cases (27.7) versus controls (27.3) (t = 3.3, *P* = 8.8 × 10^−4^).

Previous studies and case reports have documented an elevated occurrence of thrombocytopenia [22], acute renal failure [22], thrombosis [22], lymphoid malignancies [23] and hypopituitarism [24] after PUUV infection. We employed multivariate logistic regression to study the possible association of PUUV and the previously reported comorbidities. We report association with thrombocytopenia (OR = 2.46 [1.84, 3.30], *P* = 5.1 × 10^−8^), acute renal failure (OR = 1.31 [1.27, 1.35], *P* = 4.3 × 10^−6^), hypopituitarism (OR = 1.69 [1.39,2.05], *P* = 6.5 × 10^−3^), lymphoid malignancies (OR = 1.33 [1.24, 1.42], *P* = 0.02) and thrombosis (OR = 1.24 [1.19, 1.28], *P* = 0.02) overall as complications of PUUV infection (Table S2). Subsequently we employed Cox proportional-hazards models to study the temporal association of the conditions (Table S3). Associations of PUUV with thrombocytopenia (HR = 1.86 [1.34, 2.58], *P* = 2.2 × 10^−4^) and acute renal failure (HR = 1.22 [1.10, 1.34], *P* = 9.9 × 10^−5^) outcomes were supported by the Cox proportional-hazards models. In addition, temporal association with thrombosis was significant although negative (HR = 0.76 [0.63, 0.92], *P* = 4.6 × 10^−3^).

### A genome-wide association study highlights *ERAP1* and *HLA* class I and II loci

To investigate the role of single nucleotide polymorphisms (SNPs) in severe PUUV infection, we conducted a genome-wide association study (GWAS) using a dataset comprising 3650 individuals diagnosed with Haemorrhagic fever with renal syndrome (ICD A98.5) sourced from FinnGen Release 12. Of these cases, 2227 (61%) were admitted to hospital and grouped to severe cases whereas 1423 were classified as non-severe cases. We performed two GWAS analyses for severe cases, one using the remaining participants in the FinnGen cohort (= population control, N = 498 175) and another using non-severe HFRS cases as controls.

**Figure 2:**
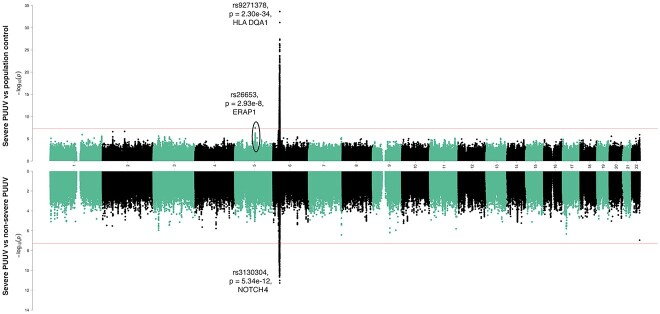
Miami plot for severe hemorrhagic fever with renal syndrome due to Puumala virus (PUUV) using population control (upper, N = 498 175) and mild cases as controls (lower, N = 1423). The X-axis represents the chromosomal position for each variant. The Y-axis shows the −log_10_(P)-value. The horizontal line indicates the genome-wide significance threshold of *P* = 5 × 10^−8^.

We identified genetic variants at the *ERAP1* gene in chromosome 5 and the HLA region in chromosome 6 with genome-wide significant associations (*P* < 5 × 10^−8^) with severe PUUV infection using population control (Fig. 2, Fig. S1, Fig. S3, Fig. S4, Table S4). The lead variant in chromosome 5 was a missense variant in the second exon of the *ERAP1* gene (rs26653, *P* = 2.9 × 10^–8,^ beta = −0.18, AF = 0.71) changing amino acid arginine in position 127 to proline. We further evaluated the association of ten most common *ERAP1* haplotypes on severe PUUV infection (Tables S6 and S7) and tested their association with hantavirus infections. We observed a consistent association with two haplotypes at the *ERAP1* locus.

The lead variant in chromosome 6 (rs9271378, *P* = 2.3 × 10^−34^, beta = 0.36, AF = 0.41) was located at the HLA class II region between *HLA-DRB1* and *HLA-DQA1*. Additionally, conditional GWAS analysis adjusting with the HLA class II lead variant, rs9271378, showed a second genome-wide significant independent association at the HLA class I region closest to the *HLA-C* gene (rs2844614, *P* = 1.0 × 10^−13^, beta = 0.31, AF = 0.13).

To explore if the same genetic factors contribute to non-severe PUUV infection and severe PUUV infection requiring hospital treatment, we compared severe PUUV cases to non-severe PUUV cases. The non-severe cases had a laboratory-confirmed PUUV infection but did not require hospital treatment. Similar to the severe PUUV versus population control GWAS analysis, we identified a genome-wide signal at the HLA region (Fig. 2, Figs S2 and S3, Table S3). The lead variant was closest to *NOTCH4* (rs3130304, *P* = 5.3 × 10^−12^, beta = 0.46, AF = 0.19), and located approximately 200 kilobases from the HLA class II region. We also observed an association with *ERAP1* lead variant rs26653 (*P* = 8.3 × 10^−5^, beta = −0.22).

Furthermore, we evaluated whether the association signal between the lead variants in severe PUUV vs population control and severe PUUV vs non-severe PUUV are independent from each other. We first examined the LD between rs9271378 and rs3130304 (R^2^ = 0.0743). We then computed association statistics for severe PUUV versus population control adjusting with the variant rs9271378. The association signal for rs3130304 was decreased from *P* = 1.01 × 10^−23^ (from non-conditioned GWAS summary statistics) to *P* = 5.84 × 10^−8^ and beta from 0.38 to 0.22. Overall, these analyses suggest that a subset of the signal may be shared between rs9271378 and rs3130304.

### HLA fine-mapping identifies a strong association with HLA-C*07:01

The HLA region is known for its high genetic diversity and many variants are in strong linkage disequilibrium (LD) with each other. To understand the complexity of genetic associations, identify the specific causal HLA alleles, and gain a deeper understanding of the biological mechanisms influencing the PUUV infection, we performed fine-mapping of the HLA region.

**Figure 3:**
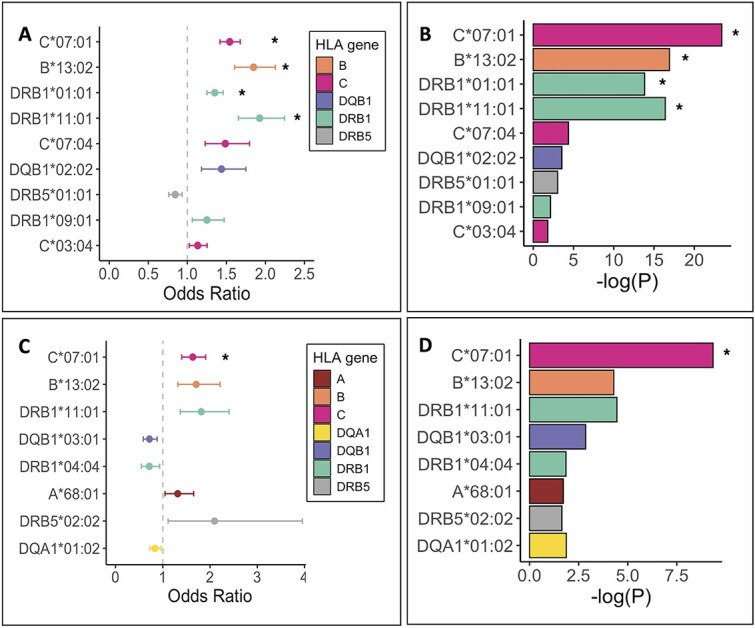
HLA fine-mapping in severe Puumala virus infection. Independent associations of HLA alleles with Puumala virus (PUUV) in FinnGen (*P*-value < 0.05). Stepwise logistic regression was performed by sequentially adding the most strongly associated HLA allele as a covariate to the multivariate regression analysis until no significant alleles remained. The graphs present alleles in order: The uppermost bar represents the allele that appeared as the most significant before adjusting with HLA alleles. We show odds ratios (A) and p-values (B) from fine-mapping of severe cases using population control and odds ratios (C) and p-values (D) from fine-mapping of severe cases using non-severe cases as controls. Asterisks after bars indicate genome-wide significant signals (*P*-values < 5 × 10–8).

We used imputed HLA allele information to assess whether HLA alleles were associated with susceptibility to PUUV infection and severity. We included alleles with minor allele frequency greater than 1% in the fine-mapping analysis (Supplementary Excel Table 1). We used multivariate logistic regression and adjusted the analysis for age at death or the end of follow-up, sex, and population structure including the first 10 genetic principal components. We found 46 associations with HLA alleles (*P* < 0.05) and 17 associations at genome-wide significant level (*P* < 5 × 10^−8^). Due to high linkage disequilibrium (LD) in the HLA region, not all associations were expected to be independent. We performed a stepwise logistic regression model to identify the individually associated HLA alleles, adjusting for the most strongly associated HLA allele until the lead variant was not statistically significant (*P* ≥ 0.05). We found four genome-wide significant (*P* < 5 × 10^−8^) HLA alleles associated with severe PUUV, all with predisposing effects (Fig. 3A and B, Supplementary Excel Tables 2 and 3). Using hospitalized individuals as cases and non-hospitalized individuals as controls, we identified 25 associations (*P* < 0.05) at the first fine-mapping round with two of them being genome-wide significant (*P* < 5 × 10^−8^). With stepwise logistic regression, one of the variants was found to be independent and genome-wide significant (*P* < 5 × 10^−8^) with predisposing effect. (Fig. 3C and D, Supplementary Excel Tables 4 and 5).

The strongest association for severe PUUV infection were with HLA alleles C*07:01 (OR = 1.54 [1.42, 1.68], *P* = 4.0 × 10^−24^), B*13:02 (OR = 1.85 [1.61, 2.13], *P* = 1.2 × 10^−17^), DRB1*01:01 (OR = 1.35 [1.25, 1.46], *P* = 1.5 × 10^−14^) and DRB1*11:01 (OR = 1.93 [1.65, 2.24], *P* = 4.1 × 10^−17^) in HLA fine-mapping analysis using population control. When assigning severe PUUV infection as cases and non-hospitalized individuals as controls the strongest associations was with C*07:01 (OR = 1.63 [1.40, 1.91], *P* = 4.6 × 10^−10^).

In addition to stepwise conditional analysis using only HLA alleles, we conducted a conditional analysis with classical HLA alleles in the SNP-level analysis (GWAS) as well as HLA-level analysis with variant data. We proceeded sequentially by adding the most significant association (HLA allele or lead variant) from each round as a covariate to the following analysis.

We identified following independent genome-wide significant (*P* < 5 × 10^−8^) associations for severe PUUV versus population control: rs9271378 (*P* = 2.3 × 10^−34^), HLA C*07:01 (*P* = 3.4 × 10^−14^) and HLA B*13:02 (*P* = 4.3 × 10^−11^). Moreover, HLA C*07:01 and HLA B*13:02 show stronger associations than our second independent associated variant rs2844614 that did not show significance after conditioning with rs9271378 and HLA C*07:01. For severe versus non-severe PUUV, the following analysis suggests rs3130304 (*P* = 5.3 × 10^−12^) as the lead signal and stronger than HLA C*07:01 and likely explains the genome-wide signal at the HLA region.

## Discussion

Here we performed a GWAS to understand host factors and genetic variants that affect the severity of PUUV infection. Utilizing data from the FinnGen cohort with over 2000 severe cases we identified genetic associations from the HLA locus and *ERAP1* gene to severe PUUV infection. Our lead variant in the *ERAP1* gene was a missense variant changing the amino acid arginine to proline. Furthermore, in the HLA region, we showed independent associations with PUUV severity in both class I and class II regions. With conditional fine mapping, we further associated the severe disease with several HLA alleles, the most significant of which were HLA*C:07:01, B*13:02, DRB1*01:01, and DRB1*11:01 alleles.

Both the HLA region and *ERAP1* gene are essential for the proper functioning of the adaptive immune response. The HLA region, located in chromosome 6, is exceptionally diverse encoding a vast array of cell surface proteins critical for immune function. These HLA molecules are responsible for presenting antigens to T cells and initiating immune responses against pathogens. Due to their polymorphic nature, variations in HLA genes profoundly impact disease susceptibility and severity in a number of infections [25].

The *ERAP1* gene, in turn, encodes a protein called endoplasmic reticulum aminopeptidase 1, which acts within the endoplasmic reticulum cleaving diverse viral and bacterial proteins into short peptides, facilitating their recognition by the immune system. Peptides are then transported to the cell surface where they bind to HLA class I molecules, HLA A, HLA B and HLA C proteins, which present viral peptides to the immune system. If the immune system recognizes the peptides as foreign, such as viral or bacterial peptides, it responds by triggering the infected cell to self-destruct [26].

Previous studies have investigated the potential role of HLA genetic variation in determining susceptibility to PUUV infection and the severity of HFRS. These studies have established a connection between several HLA alleles and haplotypes and the severe course of PUUV [7–10]. Our study is the first GWAS on PUUV infection and we strongly confirm the association to HLA region with two independent variants, one located in HLA class I region closest to the *HLA-C* gene and the other in HLA class II region in between *HLA-DRB1* and *HLA-DQA1* genes. The association at the *ERAP1* locus is a novel finding in the context of PUUV infection yet the gene has previously been linked to various autoimmune disorders such as ankylosing spondylitis and psoriasis, as well as infections including hepatitis C, influenza, and HIV [27]. Based on our findings we hypothesize that variation in the *ERAP1* gene can improve its capability to process PUUV proteins into peptides of suitable length leading to effective presentation on HLA class I molecules, a key step for functional adaptive immune response. In addition to HLA genes, it is possible that non-HLA genes also contribute to PUUV infections.

While the extended HLA region comprises over 200 genes, the majority of association signal is attributed to HLA class I or class II genes and the individual HLA alleles [28]. Previous research on PUUV genetics has relied on case–control studies and mostly focused on the association between specific HLA alleles and haplotypes and the risk of either mild or severe disease [7–10]. However, the implicated alleles have originated from relatively small studies with different alleles associated within each study. In genetic datasets, allelic associations are typically estimated by first imputing classical HLA alleles and then performing regression. Therefore, to understand which genes and alleles contribute to PUUV infection, we performed fine mapping of HLA alleles, revealing the strongest association at HLA C*07:01 followed by significant associations with B*13:02, DRB1*01:01, and DRB1*11:01. Furthermore, we demonstrated the importance of the HLA-C*07:01 allele when adjusting our GWAS analysis for the lead allele (rs9271378) showing attenuated association in the HLA class II region. To conclude, our fine-mapping analysis indicates four predisposing HLA alleles for severe disease. In addition to HLA genes, it is possible that non-HLA genes also contribute to PUUV infections.

Finally, we also show that the FinnGen sample that covers 10% of the Finnish population gives a representative picture of PUUV infection. We demonstrated that incidence rates vary in cycles of 3–4 years following the changes in vole population density as known from previous research. Peak months for incidences are summer and autumn months. We also showed that men are slightly overrepresented in the cases as well as a higher BMI is associated with the disease. We studied epidemiological associations with previously reported diseases associated with PUUV using multivariate linear regression and Cox models and found statistically significant correlations to thrombocytopenia and acute kidney renal failure, two symptoms commonly linked to PUUV infections.

Our study, the first to utilize large-scale genetic datasets to study PUUV infection, provides a comprehensive view of the disease’s genetic underpinnings. Individual sequencing data and HLA allele information enable us to employ genetic tools and methods for reliable, unbiased analysis. PUUV infections are prevalent in Finland, with diagnoses verified through laboratory testing, providing ideal conditions for investigating PUUV genetics. Nonetheless, our study has some limitations. Our analyses were exclusively performed within the FinnGen study and thus only contained individuals of Finnish ancestry. For this reason, we cannot say whether the results are generalisable to other ethnicities, other than Europeans. A replication analysis to confirm the results would be beneficial but due to low case numbers in countries with available genetic biobank data we were unable to perform such replication. We hope that future infectious disease efforts can elucidate PUUV infection genetics in large cohorts outside Finland. Additionally, in Finland, Puumala viruses cause only hemorrhagic fever with renal syndrome, whereas in other countries Hantavirus Pulmonary syndrome is a common form of the infection as well. Thus, our results are specific to HFRS, and corresponding studies for HPS would be highly beneficial. Lastly, our epidemiological analysis would benefit from a larger sample size, as FinnGen covers only about 10% of Finns, and we were unable to replicate some previous findings identified using nationwide registries. It is also noteworthy that the FinnGen dataset is enriched for disease endpoints due to the relatively high median age of participants and the significant proportion of hospital-based recruitment [11].

Our genetic study on PUUV implicates peptide processing and presentation relevant to severe PUUV infection. *ERAP1* gene polymorphism and alleles from both HLA class I and II are associated with the severe disease, shedding light on disease mechanisms and immune defence against the Puumala virus. Overall, these findings highlight the well-known association between HLA molecules and infections, suggesting that both HLA class I and class II variation contribute to PUUV infection and finally implicate the role of *ERAP1*, a known immune mediator, also in PUUV infection.

## Supplementary Material

Supplementary_word_ddae158

PUUV_supplementary_excel_ddae158

## References

[1] Vaheri A, Henttonen H, Voutilainen L. et al. Hantavirus infections in Europe and their impact on public health. Rev Med Virol 2013;23:35–49. 22761056 10.1002/rmv.1722

[2] Kanerva M, Mustonen J, Vaheri A. Pathogenesis of Puumala and other hantavirus infections. Rev Med Virol 1998;8:67–86. 10398496 10.1002/(sici)1099-1654(199804/06)8:2<67::aid-rmv217>3.0.co;2-u

[3] Vaheri A, Henttonen H, Mustonen J. Hantavirus research in Finland: highlights and perspectives. Viruses 2021;13:1452. 34452318 10.3390/v13081452 PMC8402838

[4] Makary P, Kanerva M, Ollgren J. et al. Disease burden of Puumala virus infections, 1995–2008. Epidemiol Infect 2010;138:1484–1492. 20109263 10.1017/S0950268810000087

[5] Mustonen J, Vaheri A, Pörsti I. et al. Long-term consequences of Puumala hantavirus infection. Viruses 2022;14:598. 35337005 10.3390/v14030598 PMC8953343

[6] Latronico F, Mäki S, Rissanen H. et al. Population-based seroprevalence of Puumala hantavirus in Finland: smoking as a risk factor. Epidemiol Infect 2018;146:367–371. 29310747 10.1017/S0950268817002904 PMC9134512

[7] Mustonen J, Partanen J, Kanerva M. et al. Genetic susceptibility to severe course of nephropathia epidemica caused by Puumala hantavirus. Kidney Int 1996;49:217–221. 8770970 10.1038/ki.1996.29

[8] Mustonen J, Partanen J, Kanerva M. et al. Association of HLA B27 with benign clinical course of nephropathia epidemica caused by Puumala hantavirus. Scand J Immunol 1998;47:277–279. 9519867 10.1046/j.1365-3083.1998.00302.x

[9] Korva M, Saksida A, Kunilo S. et al. HLA-associated hemorrhagic fever with renal syndrome disease progression in slovenian patients. Clin Vaccine Immunol 2011;18:1435–1440. 21775516 10.1128/CVI.05187-11 PMC3165220

[10] Ferrer C, Vial C, Ferrés G. et al. Genetic susceptibility to Andes hantavirus: association between severity of disease and HLA alíeles in Chilean patients. Rev Chilena Infectol 2007;24:351–359. 17989838 10.4067/s0716-10182007000500001

[11] Kurki MI, Karjalainen J, Palta P. et al. FinnGen provides genetic insights from a well-phenotyped isolated population. Nature 2023;613:508–518. 36653562 10.1038/s41586-022-05473-8 PMC9849126

[12] Terveyden ja hyvinvoinninlaitos . *Tartuntatautirekisterin tilastotietokanta - tapaukset - THL kuutio- ja tiivistekäyttöliittymä*. THL, 2024. https://sampo.thl.fi/pivot/prod/fi/ttr/cases/fact_ttr_cases.

[13] Ritari J, Hyvärinen K, Clancy J. et al. Increasing accuracy of HLA imputation by a population-specific reference panel in a FinnGen biobank cohort. NAR Genom bioinform 2020;2:lqaa030. 10.1093/nargab/lqaa030 PMC7671345 33575586

[14] Turner S . qqman: an R package for visualizing GWAS results using Q-Q and Manhattan plots. J Open Source Softw 2018;3:731.

[15] Barrett T, Dowle M, Srinivasan A. et al. data.table: Extension of 'data.frame', 2024. R package version 1.15.99, https://Rdatatable.gitlab.io/data.table, https://github.com/Rdatatable/data.table, https://r-datatable.com.

[16] Wickham H, François R, Henry L. et al. dplyr: A Grammar of Data Manipulation, 2023. R package version 1.1.4, https://github.com/tidyverse/dplyr, https://dplyr.tidyverse.org.

[17] Wickham H, Averick M, Bryan J. et al. Welcome to the Tidyverse. J Open Source Softw 2019;4:1686.

[18] Goel MK, Khanna P, Kishore J. Understanding survival analysis: Kaplan-Meier estimate. Int J Ayurveda Res 2010;1:274–278. 21455458 10.4103/0974-7788.76794 PMC3059453

[19] Gelfman S, Moscati A, Huergo SM. et al. A large meta-analysis identifies genes associated with anterior uveitis. Nat Commun 2023;14:7300. 37949852 10.1038/s41467-023-43036-1 PMC10638276

[20] Purcell S, Neale B, Todd-Brown K. et al. PLINK: a tool set for whole-genome association and population-based linkage analyses. Am J Hum Genet 2007;81:559–575. 17701901 10.1086/519795 PMC1950838

[21] Sane J, Ollgren J, Makary P. et al. Regional differences in long-term cycles and seasonality of Puumala virus infections, Finland, 1995–2014. Epidemiol Infect 2016;144:2883–2888. 27113030 10.1017/S0950268816000765 PMC9150413

[22] Rasche FM, Uhel B, Krüger DH. et al. Thrombocytopenia and acute renal failure in Puumala hantavirus infections. Emerg Infect Dis 2004;10:1420–1425. 15496243 10.3201/eid1008.031069 PMC3320406

[23] Kääriäinen S, Ollgren J, Dub T. et al. Risk of lymphoid malignancies increased after Puumala virus infection in Finland, 2009-2019: a retrospective register-based cohort study. Int J Inf Dis 2023;131:1–6. 36948450 10.1016/j.ijid.2023.03.026

[24] Mäkelä S, Jaatinen P, Miettinen M. et al. Hormonal deficiencies during and after Puumala hantavirus infection *Eur*. J Clin Microbiol Infect Dis 2010;29:705–713. 10.1007/s10096-010-0918-y 20397036

[25] Choo SY . The HLA system: genetics, immunology, clinical testing, and clinical implications. Yonsei Med J 2007;48:11–23. 17326240 10.3349/ymj.2007.48.1.11 PMC2628004

[26] Chang SC, Momburg F, Bhutani N. et al. The ER aminopeptidase, ERAP1, trims precursors to lengths of MHC class I peptides by a “molecular ruler” mechanism. Proc Natl Acad Sci USA 2005;102:17107–17112. 16286653 10.1073/pnas.0500721102 PMC1287962

[27] Saulle I, Vicentini C, Clerici M. et al. An overview on ERAP roles in infectious diseases. Cells 2020;9:720. 32183384 10.3390/cells9030720 PMC7140696

[28] Klein J, Sato A. The HLA system. First of two parts. N Engl J Med 2000;343:702–709. 10974135 10.1056/NEJM200009073431006

